# The evaluation of comprehensive medication management for chronic diseases in primary care clinics, a Texas delivery system reform incentive payment program

**DOI:** 10.1186/s12913-020-05537-3

**Published:** 2020-07-20

**Authors:** Tong Han Chung, Ricardo J. Hernandez, Anaelle Libaud-Moal, Linh K. Nguyen, Lincy S. Lal, J. Michael Swint, Pio Juan Lansangan, Yen-Chi L. Le

**Affiliations:** 1 Department of Healthcare Transformation Initiatives, McGovern Medical School, The University of Texas Health Science Center at Houston, 1200 Binz Street, Suite 730, Houston, TX 77004 USA; 2 Division of Cardiology, Department of Internal Medicine, McGovern Medical School, University of Texas Health Science Center at Houston, 6431 Fannin Street, Houston, TX 77030 USA; 3 INSERM (Institut National de la Santé et de la Recherche Médicale), 63 Quai Magellan 44021, Nantes Cedex 01, France; 4 Department of Management, Policy and Community Health, School of Public Health, University of Texas Health Science Center at Houston, 1200 Pressler Street, Houston, TX 77030 USA

**Keywords:** Comprehensive medication management, Drug therapy problem, Cost analysis

## Abstract

**Background:**

The Institute of Medicine reported that more than 1.5 million preventable adverse drug events occur annually in the United States. Comprehensive Medication Management (CMM) is the medication review process to improve clinical outcomes, enhance patient adherence, reduce drug therapy problems and reduce health care costs. University of Texas (UT) Physicians implemented a CMM program in several community-based clinics. We evaluated the effectiveness of CMM to reduce drug therapy problems and achieve medical cost savings.

**Methods:**

This was a retrospective, observational study of CMM participants from October 2015 to September 2016. Program participants included patients aged 18 years or older who had taken more than 4 prescribed medications and were diagnosed with at least one of the following chronic diseases: hypertension, congestive heart failure, chronic obstructive pulmonary disease, asthma or diabetes. Under the CMM program, a clinical pharmacist reviewed patients’ electronic health records and created action plans to resolve identified drug problems. As part of the evaluation of the clinical process, two independent physicians conducted peer review on the recommendations issued by the pharmacist in order to establish inter-rater reliability of drug therapy problems and potential consequent medical services. The drug therapy problems were identified and classified into four categories: indication, effectiveness, safety and/or compliance. The average cost of avoided medical services was obtained based on cost extrapolations from the literature, combined with hospital discharge data. Potential medical services avoided were linked to the average cost of those services to calculate the total cost savings of the program from the payers’ perspective.

**Results:**

By reviewing electronic health records of 3280 patients, the pharmacist identified 301 drug therapy problems and resolved 49.8% of these problems with collaboration from the patient’s primary care physician or care team. The most commonly identified drug problems were related to potentially adverse drug reactions or inappropriate drug dosage. The CMM program resulted in potential cost savings of $1,143,015.

**Conclusions:**

The CMM program resolved medication therapy problems among program participants and achieved significant health care cost savings.

## Background

Medication-related problems, such as inappropriate medication use and adverse drug events that are potentially preventable, can lead to avoidable hospital and emergency department admissions, as well as unnecessary healthcare costs. In 2006 the Institute of Medicine reported that the annual number of preventable adverse drug events in the United States exceeded 1.5 million [1]. In 2000, the estimated annual cost of medication-related problems in the U.S. was $177.4 billion [2].

Comprehensive Medication Management (CMM) is a medication review process aimed at assessing a patient’s medication regimen and optimizing medication therapy [3]. This evidence-based program can result in improved clinical outcomes and lowered health care costs. For instance, a CMM program implemented in Connecticut resulted in an estimated annual reduction of $1123 in medication claims per patient by reducing unnecessary medications and changing to less expensive medications [4]. CMM for patients with chronic conditions resulted in a significant reduction in Emergency Department (ED) visits, with a cost savings of $2.10 to $2.60 for every $1.00 spent [5]. A study of diabetic patients found that CMM significantly reduced average Hemoglobin A1c level and reduced total health care cost by $1031 per patient using pre and post comparison [6]. A study of CMM service in a patient-centered medical home showed that more than 70% of patients with an uncontrolled chronic condition had improved clinical outcomes, and the potential estimated cost avoidance was about $1.9 million annually [7].

Under the Section 1115 of the Social Security Act (Medicaid 1115 Waiver), approved by Centers for Medicare and Medicaid Services (CMS) for Texas in December 2011, the Texas Delivery System Reform Incentive Payment (DSRIP) program was created. Twenty Regional Healthcare Partnerships received incentive payments to develop programs to improve access to health care, the quality of care, and the cost-effectiveness of care for its patients, with a focus on underserved populations. Under this waiver, UT Physicians implemented many programs including care coordination and CMM in its primary care clinics in 2015. UT Physicians is the practice plan of the UT Health McGovern Medical School. It has over 100 clinics in Houston, Texas and the surrounding areas.

UT Physicians’ care coordination program is a collaboration between providers and non-physician staff, including clinical case managers, community health workers, social workers, health educators, clinical pharmacists, and diabetes educators, to improve the health of patients. Care coordination services are tailored to the patients’ health care needs as care teams follow up with patients who missed appointments, provide regular disease self-management education, and refer patients to community-based resources, specialists, and counseling when necessary. The CMM program provides specific care coordination services to optimize medication use, identify and resolve drug therapy problems, and follow-up with providers and the care team on health outcomes [8]. Specific to CMM, certain care team members, such as the case manager or community health worker, may help eligible patients enroll in the CMM program while other care team roles, such as the clinical pharmacist, would either work directly with the patient and/or the patient’s primary care provider to conduct CMM and related-services.

The objective of this study is to evaluate the effectiveness of this CMM program in decreasing drug therapy problems and related medical costs.

## Methods

### CMM program and populations

UT Physicians implemented the CMM program in its community-based clinics to improve the health of patients with chronic conditions by providing care coordination and management under the patient-centered medical home. One goal of CMM is to reduce potential medication errors by both patients and healthcare providers. The program focuses on serving polypharmacy patients with a diagnosis of one or more chronic diseases because polypharmacy patients are more prone to misuse their medications and to develop adverse effects from prescription drug interactions.

**Fig. 1:**
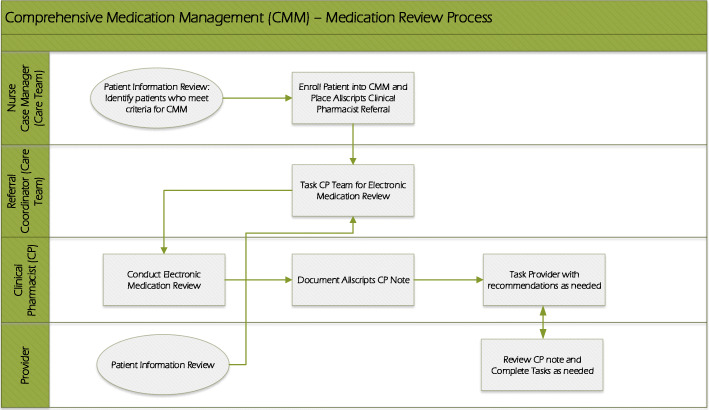
Comprehensive Medication Management Flowchart

Inclusion criteria for this program are patients aged 18 years or older who are taking four or more prescribed medications and are diagnosed with at least one of the following chronic diseases: hypertension, congestive heart failure, chronic obstructive pulmonary disease, asthma, or diabetes. Eligible patients were recruited by a care team that includes a clinical nurse case manager and/or a community health worker. During the enrollment of the CMM, nurse case mangers notified patients about the comprehensive medication review by clinical pharmacist. All participants were referred to the clinical pharmacist by their clinical nurse case manager during pre-visit or post-visit planning, or by their primary care provider during their appointment (Fig. 1).

The goals of the CMM program are to improve medication safety, optimize medication therapy, identify and resolve medication-related issues, reduce barriers to accessing prescription medications, improve patient medication compliance and reduce medications errors. Upon implementation of the physician-pharmacist partnership at UT physicians, the CMM program services, delivered by the pharmacist, encompasses the following actions:

1. Review patient’s medical record and assess pertinent information which includes but are not limited to: allergies/reactions, height, weight, vitals labs, renal/hepatic function, problems/diagnoses, family history, social history, past surgical procedures, medications, provider visit and progress notes, provider specialist notes, discharge summaries, radiology reports
2. Evaluates all of a patient’s medical conditions and medications and include:
  - Medication optimization including care gap analysis, additional therapy, medication discontinuation, and dose adjustments per recommendations only
  - Medications with no indication
  - Polypharmacy
  - Adverse drug reactions
  - Risk mitigation strategies
  - Major drug interactions (drug-drug, drug-disease, drug-food)
  - Labs and follow-up
  - Formulary/costs
  - Immunization recommendations
  - Over-the-counter recommendations
  - Clarification to understand provider’s intention with a specific prescription
  - Referrals or follow-up with specialists (e.g. cardiologist)
  - Medication adherence issues
  - Medication reconciliation issues
  - Patient education
3. Document all patient encounters using pre-approved templates in the electronic health record
  - At the very minimum, the clinical pharmacist documents the provider’s initial patient care consult/referral order, conducts an assessment per available information, and provides a needs-matched recommended plan of care if medically necessary
  - If a provider approves of a recommended plan of care, the clinical pharmacist executes the plan of care upon approval and delegation by the provider.

Since the clinical pharmacist is part of the care team and also co-located within the clinics, the clinical pharmacist has access to the patient’s medical information and could reasonably verify an appropriate referral by the clinical nurse case manager. In order to provide the CMM program to multiple UT Physicians clinics with one clinical pharmacist, the CMM program operates as a centralized service. Face-to-face consult services about medication and its therapeutic plan are conducted at specific clinics per provider or patient request.

Through CMM, the clinical pharmacist identified drug therapy problems such as indication, effectiveness, safety or adherence, and developed an action plan to resolve each identified problem. The pharmacist’s proposed action plans and recommendations concerning each drug therapy problem were sent to each patient’s physician as a clinical pharmacist note using the messaging system in Allscripts, an electronic health records application. Then the pharmacist followed up with the physician in about 3 weeks via the internal messaging system to ensure recommendations were reviewed and that the provider implemented changes (Fig. 1). If a recommendation was deemed urgent or of high importance, the pharmacist would directly contact the provider by phone.

Implementation of the pharmacist’s recommendations was verified as ‘accepted’, ‘partial’, or ‘declined’: ‘accepted’ means the provider and patient fully adopted the recommendations for a change in treatment; ‘partial’ means provider followed up the recommendations after discussing with the pharmacist or ordered lab test, but did not change the medication treatment based on the clinical decision; ‘declined’ means provider did not take action regarding the recommendations. If the pharmacist’s recommendation was ‘accepted’, physician informed the patients about such changes in the treatment plan provided by the clinical pharmacist.

### Study design

A retrospective observational study was performed among patients seen in the CMM program from October 2015 to September 2016. We used comprehensive medication review notes and electronic medical records data. The presence of chronic disease for a patient was confirmed using the International Classification of Diseases, Ninth and Tenth Revision, Clinical Modification codes (ICD-9-CM and ICD-10-CM).

The drug therapy problems were identified and classified into indication, effectiveness, safety and/or compliance, based on the pharmacotherapy workup notes [9]. To validate the pharmacist’s conclusion and ensure study reliability, two cardiology fellows, independent of the CMM program, served as external reviewers. In this peer review process, the cardiologists reviewed cases objectively and then discussed them with the pharmacist in order to reach a consensus on the drug therapy problem and potentially avoided medical services in the short term (up to 6 months). Previous peer review assessed that the therapeutic decision made by the pharmacist was reliable [10]. Using a structured implicit review process, a peer review panel evaluated the therapeutic decisions, including the drug therapy problem, action to resolve the problem, condition status evaluation, and estimate of cost savings by the pharmacists. The evaluation decision from the peer review panel showed a 97.9% clinically credible level of agreement with respect to the pharmacist’ determinations [10].

### Cost estimations

The potential medical services avoided, including outpatient clinic visits, specialty office visits, emergency room visits, and hospitalizations associated with resolved drug therapy problems, were projected by the clinical pharmacist and reviewed by the two cardiologists in order to get a consensus. Each drug therapy problem can be associated with multiple potential medical services. Drug therapy problem and the medical service avoided were captured using the medication review note.

The Texas Health Care Information Collection (THCIC) inpatient and outpatient discharge data file for the first quarter of 2014 was used to calculate the average charges of emergency room (ER) visits and hospitalizations. The average charges of avoided ER visits and hospitalizations were calculated for hospitals in Waller, Fort Bend, and Harris counties, where the service area is located. The service area was defined as census tracts within a 7-mile radius of UT Physicians clinic locations. Hospital charges were converted to costs using hospital-specific inpatient cost-to-charge ratio (CCR) for Medicaid in March 2014 from the Texas Health and Human Services Commission [11]. If hospital-specific CCRs were missing, the mean CCR for all hospitals was utilized. Since THCIC only has outpatient surgical and radiological service charges from hospitals and ambulatory surgery centers, the average costs of outpatient clinic visits and specialty office visits were obtained from the Fairview Health Service study and adjusted for inflation [12].

The wholesale prices of the drugs were obtained from UpToDate® and the website for ‘Nature Made’ products. To estimate the cost savings of reducing/increasing or changing the drug product, the drug cost differences for patient between prior and post CMM were estimated.

To calculate the total cost savings by CMM, the number of medical services avoided was linked to the average cost of each medical service. Cost savings by CMM was estimated from October 2015 to September 2016. To reflect the direct impact of the CMM program, only short-term cost savings, resulting from avoided events, was estimated. Cost savings were calculated from the payer’s perspective and adjusted for inflation using the medical consumer price index (CPI). Costs were estimated in 2016 dollars.

Potentially there can be multiple avoided medical services per identified drug problem, which creates uncertainty in the cost saving calculation. For example, a medication problem can lead to additional outpatient visits and/or a hospital admission. A scenario-based sensitivity analysis was conducted based on two hypothetical scenarios: 1) only one most severe medical service per medication problem was avoided and 2) only one least severe medical service per medication problem was avoided. The hierarchical order of medical service was based on the consequent severity of illness. For example, hospitalization would be considered more severe than an outpatient visit. Statistical analysis was conducted using SAS version 9.4 (SAS Institute, Inc., Cary, North Carolina, USA). This study was approved by the Institutional Review Board at the University of Texas Health Science Center at Houston.

## Results

The clinical pharmacist reviewed the electronic health records of 3280 patients. Table 1 shows that most patients were white (59.79%), female (61.65%), and 40 years old or older (93.57%). Approximately 48% of patients had two or more chronic diseases. Among the 3280 patients reviewed, the pharmacist initially identified 311 drug therapy problems among 290 patients. After discussions with external the physician reviewers, there were 301 agreed upon drug therapy problems among 280 patients (8.54%) (Table 1).

**Table 1:** Characteristics of chronic disease patients seen in CMM, 2015–2016

Patient Characteristics	Number (***N*** = 3280) (%^a^)
*Gender*	
Male	1258 (38.35%)
Female	2022 (61.65%)
*Race*	
White	1961 (59.79%)
Black	605 (18.45%)
Other	714 (21.77%)
*Age*	
18–39 years	211 (6.43%)
40–64 years	1559 (47.53%)
65–79 years	1161 (35.40%)
80+ years	349 (10.64%)
*Number of Chronic Disease(s)*	
1	1704 (51.95%)
2	1283 (39.12%)
3	253 (7.71%)
4	38 (1.16%)
5	2 (0.06%)
*Number of drug therapy problem(s)*	
0	3000 (91.46%)
1	260 (7.93%)
2	19 (0.58%)
3+	1 (0.03%)
*Insurance*	
Medicaid	266 (8.11%)
Medicare	1367 (41.68%)
Managed Care	1594 (48.60%)
Others (Self Pay/Indemnity)	53 (1.62%)

^a^ Percentages may not add up to100 due to rounding

Of the 301 drug therapy problem classified, the most common problems were related to overall “safety” (56.8%, *n* = 171), with the “adverse drug reaction” sub-category containing 130 problems. The second most common drug problem was related to “indication”, with a total of 105 (34.9%) associated problems in the categories of “unnecessary drug therapy (*n*=55)” or “needs additional drug therapy (*n*=50)” (Table 2).

**Table 2:** Drug therapy problems

	Category of drug therapy problems	Number of drug therapy problems (***N*** = 301) (%^a^)
Indication	Unnecessary drug therapy	55 (18.27%)
	Needs additional drug therapy	50 (16.61%)
Effectiveness	Ineffective drug	2 (0.66%)
	Dosage too low	19 (6.31%)
Safety	Adverse drug reaction	130 (43.19%)
	Dosage too high	41 (13.62%)
Adherence	Non-adherence	4 (1.33%)

^a^ Percentages may not add up to 100 due to rounding

When the pharmacist identified a potential drug problem, he would communicate the drug issue(s) to the patient’s physician. In general, the patient’s physician would take action, based on the pharmacist’s recommendations. ‘Accepted’ and ‘partial’ implementation rates of recommendations by physicians were 49.8% (*n* = 151 identified drug therapy problems) and 42.8% (*n* = 129 identified drug therapy problems), respectively.

In total, 270 medical services associated with 150 ‘accepted’ implementations were potentially avoided with a potential cost savings of $1,185,610 (Table 3). The largest number of medical services avoided were related to clinic outpatient visits (*n* = 131 events), followed by reduced/increased drug products (*n* = 126 events), emergency room visits (*n* = 66 events), hospital admissions (*n* = 62 events), and specialty office visits (*n* = 11 events). The highest cost savings per event was among hospital admissions avoided at $17,263 cost per event for a total savings of $1,070,306. Avoided hospital and ED visits accounted for over 90% of cost savings. Therefore, 90% of the cost savings is accounted for by only 40% of the avoided events.

**Table 3:** Estimated cost savings in drug therapy management program

Potential Medical Service(s) Avoided	Number of Events	Estimated Cost per Event (2016 US dollars)	Total Savings
Clinic outpatient visit	131	$206	$26,986
Specialty office visit	11	$264	$2904
Emergency room visit	66	$1021	$67,386
Hospital admission	62	$17,263	$1,070,306
Reduce/Increase drug product	126	Varies and was calculated based on average wholesale price of the drugs	$18,028
**Total**	**396**		**$1,185,610**

The scenario-based sensitivity analysis showed that the total cost savings ranged from $48,077 to $1,106,426 for the CMM program focusing on only one potentially avoided medical service (Table 4). For hospital admissions, the highest cost saving scenario, the avoided medical service yielded in excess of 1 million dollars in cost savings. For clinic outpatient visits, the lowest cost saving scenario, the avoided medical service potentially saved $26,986 (Table 4).

**Table 4:** Scenario-based sensitivity analysis

Potential Medical Service(s) Avoided	Number of Events	Estimated Cost per Event (2016 US dollars)	Total Savings
**The highest savings scenario**			
Clinic outpatient visit	68	$206	$14,008
Specialty office visit	0	$264	$0
Emergency room visit	4	$1021	$4084
Hospital admission	62	$17,263	$1,070,306
Reduce/Increase drug product	126	Varies and was calculated based on average wholesale price of the drugs	$18,028
**Total**	**260**		**$1,106,426**
**The lowest savings scenario**			
Clinic outpatient visit	131	$206	$26,986
Specialty office visit	0	$264	$0
Emergency room visit	3	$1021	$3063
Hospital admission	0	$17,263	$0
Reduce/Increase drug product	126	Varies and was calculated based on average wholesale price of the drugs	$18,028
**Total**	**260**		**$48,077**

## Discussion

This study evaluated the outcomes of CMM among 3280 patients at community-based primary care clinics over a one-year period. The CMM program resolved drug therapy problems among program participants and achieved significant potential health care cost savings. These results are consistent with previous research findings that CMM reduces drug therapy problems and saves health care costs [4–7]. Our results indicate the resolution of 49.8% of identified problems and the avoidance of nearly 300 avoided medical services that would have potentially occurred if the identified problems were left unresolved. This resolution rate may be attributed to the collaborative discussions of drug therapy problems between external reviewers and the pharmacist, which in turn may have enhanced the acceptance rate of the physicians. The cooperative follow-ups between pharmacist and physicians were also helpful to increase the resolution rate. Our results indicate that the CMM had a favorable impact on quality of care with regards to avoided medical services and potential cost savings due to resolved drug therapy problems. Consequently, implementation of CMM could play an important role in the upcoming value-based purchasing models that are likely the future direction of healthcare.

The results of the scenario-based sensitivity analysis showed that the cost savings varied considerably according to the potentially avoided medical services. These results are similar to those of previous studies of CMM programs, which also show positive returns-on-investment [4–6, 12]. However, the return-on-investment calculations used in the other studies were conducted only from the payers’ perspectives. Therefore, additional study of the return-on-investment models using actual medical service changes from the provider perspective may be necessary for the future sustainability of the program at clinic or provider level. CMM services may be a compensable practice under the pay-for-performance or pay-for-quality programs, which could make the CMM service sustainable.

This study evaluated UT Physicians’ CMM program in its earlier stages of implementation and therefore has some limitations. The first limitation is the smaller number of drug therapy problems identified in this study compared to previous studies that examine CMM. As with any new intervention, the buy-in of providers was challenging. Providers were comfortable with the pharmacist communicating directly with the provider, but were hesitant to allow the pharmacist to have direct communication with the providers’ patients. Therefore, the pharmacist relied mainly on the medications list available in each patient’s electronic health record. As the CMM program has continued, we have been able to hire additional clinical pharmacists which has resulted in more direct pharmacist-patient communication. Without direct communication with the patient, the pharmacist would have not been able to verify if patients were also taking medications that are not represented in the medication list prescribed by providers outside of UT Physicians. As the CMM program has continued, providers are more aware of the CMM program and its benefits and therefore have allowed the clinical pharmacists to have direct communication with their patients. More clinics have also requested the CMM program so we have expanded the program and recruited additional clinical pharmacists. With additional pharmacists and provider buy-in and adoption, the CMM program has expanded to include direct patient-pharmacist communication, which will likely lead to an increased identification of medication issues.

The second limitation is the uncertainty in the estimation of potentially avoided medical services, which are primarily based on clinical judgment by the clinical pharmacist. This may in turn lead to the overestimation of costs savings compared with the savings using actual avoided medical services. However, this limitation was addressed with the use of independent reviews and discussions with two external physician reviewers to improve the validity of the result. Additionally, we also address the uncertainty issue using the scenario-based sensitivity analysis previously discussed.

The third limitation is that study results may not generalize to other diseases as our study focused on patients diagnosed with at least one of five specific chronic diseases, and only focused on healthcare costs from the study institution’s perspective. Additionally, health care institutions with different payer structures than our current institution may also have different results. That is, CMM for diseases at other institutions, with variable accessibility for patient information or diverse reimbursement structure, may have different economic results. Nevertheless, the results of our study will be helpful in guiding health care providers and policy decision makers in implementing and modifying CMM programs at primary care clinics. This potential cost saving result also supports the claim that CMM programs will have a positive financial impact on the health care system and potentially be sustainable.

## Conclusions

In conclusion, the CMM program resolved 301 drug therapy problems and in doing so prevented 270 potential medical services. Related potential cost saving was estimated to be $1,185,610 in 2016 US dollars. CMM can be helpful in saving total health care costs among chronic disease patients by detecting drug therapy problems early and preventing subsequent high cost admissions and ER visits that would result if drug therapy problems were left unresolved. This program supports DSRIP goals of improving quality of care, patient health status, and cost savings. The cost savings were realized in both Medicaid and non-Medicaid patients. However, as this study only estimated cost savings associated with medical care, future research using comprehensive claim datasets is needed to estimate both inpatient and outpatient medical and pharmacy expenses. This would provide a more complete understanding of economic outcomes of CMM in impacted populations.

## Data Availability

The datasets analyzed during the current study are not publicly available due to the protected health information, but are available from the corresponding author on reasonable request.

## Abbreviations

CMM: Comprehensive Medication Management
UT: University of Texas
DSRIP: Delivery System Reform Incentive Payment
CMS: Centers for Medicare and Medicaid Services
THCIC: Texas Health Care Information Collection
ER: Emergency room
CCR: Cost-to-charge ratio
